# Using augmented reality technology for balance training in the older adults: a feasibility pilot study

**DOI:** 10.1186/s12877-021-02061-9

**Published:** 2021-02-26

**Authors:** Sven Blomqvist, Stefan Seipel, Maria Engström

**Affiliations:** 1 Faculty of Health and Occupational Studies, University of Gävle, Gävle, Sweden; 2 Department of Information Technology, Uppsala University, Uppsala, Sweden; 3 Faculty of Engineering and Sustainable Development, University of Gävle, Gävle, Sweden

**Keywords:** Fall, Training, Postural stability, Augmented reality

## Abstract

**Background:**

Impaired balance leading to falls is common in the older adults, and there is strong evidence that balance training reduces falls and increases independence. Reduced resources in health care will result in fewer people getting help with rehabilitation training. In this regard, the new technology augmented reality (AR) could be helpful. With AR, the older adults can receive help with instructions and get feedback on their progression in balance training. The purpose of this pilot study was to examine the feasibility of using AR-based visual-interactive tools in balance training of the older adults.

**Methods:**

Seven older adults (66–88 years old) with impaired balance trained under supervision of a physiotherapist twice a week for six weeks using AR-based visual-interactive guidance, which was facilitated through a Microsoft HoloLens holographic display. Afterwards, participants and physiotherapists were interviewed about the new technology and their experience of the training. Also, fear of falling and balance ability were measured before and after training.

**Results:**

Five participants experienced the new technology as positive in terms of increased motivation and feedback. Experiences were mixed regarding the physical and technical aspects of the HoloLens and the design of the HoloLens application. Participants also described issues that needed to be further improved, for example, the training program was difficult and monotonous. Further, the HoloLens hardware was felt to be heavy, the application’s menu was difficult to control with different hand manoeuvres, and the calibration took a long time. Suggestions for improvements were described. Results of the balance tests and self-assessment instruments indicated no improvements in balance performance after AR training.

**Conclusions:**

The study showed that training with the new technology is, to some extent, feasible for the older adults, but needs further development. Also, the technology seemed to stimulate increased motivation, which is a prerequisite for adherence to training. However, the new technology and training requires further development and testing in a larger context.

**Supplementary Information:**

The online version contains supplementary material available at 10.1186/s12877-021-02061-9.

## Background

Impaired balance is a common cause of reduced health in the older adults. Impaired balance can lead to falls, reduced independence, and reduced daily activity that in turn can lead to illness and premature death [1]. Annually, 35–45% of community-dwelling people over 65 years of age and 50% of older adults report falls [2, 3], which accounts for more than half of the injuries leading to hospital care [4]. A Swedish study showed that less than half of the older adults met a physiotherapist after inpatient hospital stay. Although there was a willingness to exercise, there was uncertainty about the type of exercise to perform to regain physical ability [5]. There is strong evidence that balance training prevents falls and increases independence for the older adults [6]. It is also known that the training should include strength, balance, flexibility, endurance and include dynamic exercises; also, it should be done 2–3 times a week for three months to be effective [1, 4, 7, 8], which is consistent with a recently published meta-analysis that shows that falls rates are most affected if the exercise consists of multiple types [9].

Also to be effective, it is important that older adults have adherence to training advice [9, 10]. Most of the older adults who train at home (79%) struggle to implement fully their prescribed balance training to reduce falls [11]. New forms of exercise based on technologies such as Virtual Reality (VR) and Augmented Reality (AR) were shown to increase adherence to training protocols, and to stimulate cognitive abilities in older adults. Studies show that training should not only stimulate physical ability but also executive processes, and therefore should include enriched environments that provide physical activities decision-making since these are believed to facilitate development of both motor performance and brain functions; AR exercises have the ability to facilitate this [12].

AR provides opportunities to create new methods for training and rehabilitation with spatially properly calibrated 3D visualizations. Hopefully, AR-glasses can provide more people the opportunity for individualized training at home, which could increase adherence and create a more controlled implementation of physical training sessions. AR in various forms has been successfully used in skill training for doctors/healthcare personnel, and is considered suitable for training that requires high motor skills and careful spatial mobility [13]. Currently, studies are scarce that focus on physical training/rehabilitation for patients. An AR-training program (based on a video stream from a camera perspective) has been used as rehabilitation after stroke [14–16], as training in Parkinson’s disease [17, 18], to treat phantom pain in the arms [19], for shoulder problems [20], and as balance walking training for the older adults [21–23]. The few studies available indicate that AR in various forms alone or in combination with other methods may be an alternative to traditional training/rehabilitation.

The latest technology in AR glasses enables the creation of properly calibrated 3D visualizations in real time, which are spatially aligned with the real environment. This way of visually combining virtual 3D content that is metrically correct with the real environment is referred to as Mixed Reality (MR), and the displays facilitating this are sometimes called holographic display. The novelty in activity training of the older adults, as described herein, is that new information technology in the form of a holographic computer display (HoloLens™ glasses) is used to develop health-promoting services in the care of the older adults. More specifically, it is on the technical side of the method development to try new ways to visualize 3D instructional content spatially aligned with real (known) context to the older adults. Unlike previous applications of AR technology in healthcare (see above), which are based on the user perceiving reality as a video stream from an exocentric camera perspective, the holographic display allows the user to observe their real environment from their own perspective and thus can perceive and interact with 3D training content in a calibrated and metrically correct way.

When developing new complex interventions, the Medical Research Council (MRC) has expressed that the process needs to be systematic and use the best available evidence and appropriate theory. Then it should be tested with a thorough method. In a first stage, feasibility/piloting studies are important key elements in developing the complex intervention, and an important question then is whether it works in everyday life. This first step is to optimize its design and develop the intervention before running a full-scale study [24]. Furthermore, the MRC has recommended using both qualitative and quantitative methods when evaluating the process and the outcome [24, 25].

To stimulate training for the older adults who need to improve their balance we have developed balance training aids using AR and the HoloLens. The older adults get help to perform exercises correctly with both verbal and visual feedback. Exercise automatically increases as the person performs better and hopefully with the help of the training aid one can increase motivation and adherence to training for a better result. The balance training with the new aid is based on strong evidence of what works for the older adults, but in the present study, we only tested the new technique for six weeks since the focus was more on the feasibility of the new way of training and not on its effects. The concept of feasibility in this study refers to how feasible the training is with the help of the new technology and training approach.

The purpose of the present pilot study was to test the feasibility of AR based on a holographic display for visual-interactive activity training for older adults persons with balance impairments. The following questions were addressed: a) how do older adults people experience the new technology as an aid in balance training? b) how are the older adults people adhering to the HoloLens/app’s instructions and exercises? c) what experiences do physiotherapists have of the new technology as aids in balance training for older adults people? d) how do balance ability, fear of falls, and participants’ perception of their balance ability change over time after the training period for older adults who use the new technology as aids in balance training?

## Method

### Design

The study was a prospective study that used a mixed-method approach. Seven people trained their balance ability twice a week for six weeks using the HoloLens glasses with new training applications. Both quantitative and qualitative methods were used to assess the feasibility of the new AR-based training method. The qualitative method consisted of interviews with participants about their experiences of the new technology and training. The quantitative methods measured balance ability using both questionnaires and balance tests. Results from both the qualitative and quantitative analyses were jointly interpreted to answer the study questions.

### Participants and recruitment

The study originally included eight participants with impaired balance and two physiotherapists. One person had to cancel due to illness. The remaining seven participants consisted of two men and five women between 66 and 88 years of age, and the average age was 74 years. The physiotherapists were recruited from a health center near the university. The participants were recruited and asked by the physiotherapists if they would like to be in a study to evaluate a new technique that trained the balance ability. During the study, none of the participants had any other treatment elsewhere or received any other treatment from the physiotherapists that took part in the study. Inclusion criteria were a) older than 65, b) had been referred for treatment for impaired balance. Exclusion criteria were a) no cognitive impairments (e.g. developmental disorder or dementia) that make it difficult to participate in the study, such as unability to follow protocol, answer interview questions or complete a questionnaire. To find out, physiotherapists asked the participants, or b) functional impairment due to neurological causes, e.g. stroke and Parkinson, or c) blindness.

### Procedure

To develop the new training aid, a group was assembled that consisted of three experts in the technical application AR technology HoloLens (Microsoft), two physiotherapists with 6.5, 13 years of work experience and one physiotherapist/ first author with 29 years of theoretical and practical experience in balance training, and one expert on the care of the older adults. The training application was developed jointly in the group and was test run as well as improved based on what emerged during the test run. The two physiotherapists from the health centre practiced with the training application to become well-versed in both the training and the technical aspects. This means that they have good understanding of what the participants see and should do. The physiotherapists at the health center recruited the older adults participants to the intervention by asking those who had been referred for treatment for impaired balance if they were willing to participate in the study. None of the authors was involved in the recruitment and training of the participant. Participants were informed about the study’s purpose, and the training period began with participants answering questionnaires and being tested about their balance ability. This was done by the first author with assistance of the two physiotherapist from the health center. Then, the participants trained with the new balance training aids under supervision of the physiotherapists twice a week for 20 min each time for six weeks. Afterwards, the tests were repeated, and all participants and the two physiotherapists were interviewed about their experiences of the training and the new technology. Finally, data were compiled and analyses were done by the research team.

### Study intervention

By using the new AR technology via the HoloLens, participants can see the game-like scenario as a hologram on the lens of the glasses at the same time as they see the real world through the glasses. With only 34 degrees horizontal field of view (FoV_h_), the visual overlay created by the HoloLens display covers merely the central parts of the user’s entire visual field. The developed training application consisted of two games.

**Fig. 1:**
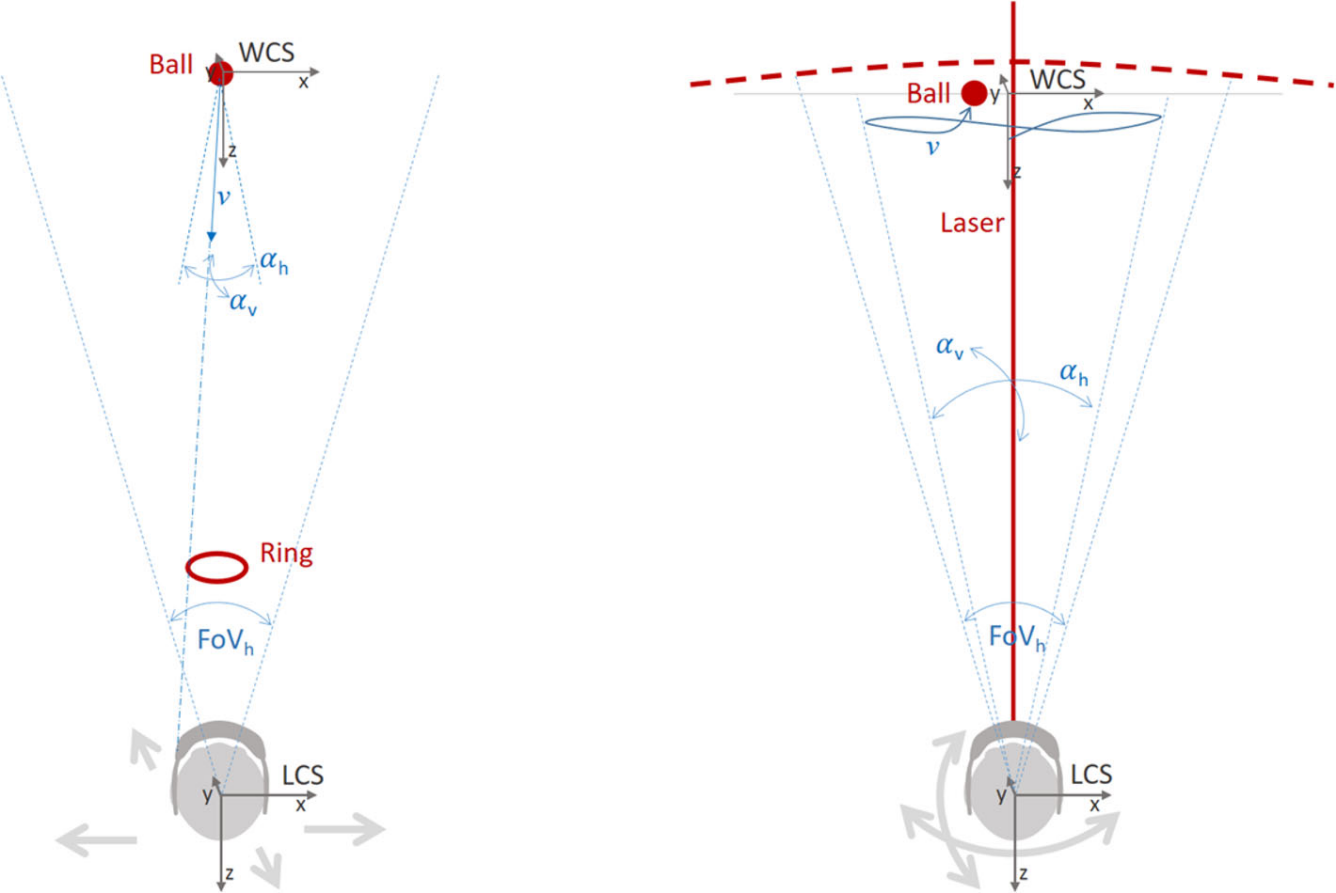
Illustration of the spatial relationship between the user and the virtual objects (red) in the two AR-based game scenarios. Game 1 is to the left and Game 2 is to the right

**Fig. 2:**
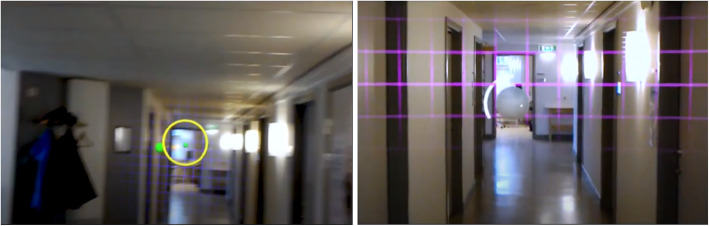
Screen captures from the HoloLens built in video camera showing how the user experiences the synthetic game elements mixed with reality in the two games. Game 1 is to the left and Game 2 is to the right

The first training game aimed to stimulate sideways motion of the upper body, and thus moving the center of gravity to the side, while remaining in a fixed position on the ground. To encourage this motion, we designed an interactive 3D task that required users to “catch” a ball using a ring. Figure 1 (left) illustrates the design and spatial relationships between the user and the virtual objects. Two coordinate systems are needed here to describe the dynamic interaction in this game scenario. The local coordinate system (LCS) of the user’s head as determined in real time by the spatial sensors of the HoloLens represents the center of the head at eye level, as well as head orientation in terms of the line of sight (negative z-axis), and roll (in terms of the x-, or y-axis). At the start of each game, a world coordinate system (WCS) is positioned 2 m in front of the user in the center of the field of view. During the game, the WCS is always locked in this position, while the LCS follows the users’ head motion and rotation. The ring with an inner radius twice the size of the ball is positioned in front of the user at a distance of 0.5 m within the LCS. Balls start from the center of the WCS flying towards the user at a constant velocity (*v)* following a linear trajectory. The direction of the linear trajectories could be randomly varied within horizontal (α_h_) and vertical (α_v_) angular ranges. The animation of the ball takes place in the static coordinate system WCS, and it is therefore independent of user’s head motion. The challenge for the user was to move the ring in front of their heads such that the ball would pass through the ring. Since the ring is anchored to the LCS, head motion (in the global xy-plane) would be required to solve this task, while the z-axes of the local and global coordinate systems should be kept in alignment to maintain a sufficiently large efficient aperture of the ring. The degree of difficulty of game 1 could be controlled by defining velocity (*v)*, as well as the angles of α_h_ and α_v_. Table 1 gives accounts of the parameters for different levels of difficulties in our study. The table also contains the time lapse between the generation of successive balls, as well as the number of balls (count) at each level. The score of the game pertains to the number of successfully caught balls in each attempt. When a participant obtained all points on current particular level of practice, the level increased. Hence, according to Table 1, with successive levels, the ball would move faster and being further out to the side. In addition to the game parameters, the difficulty level could also be increased by reducing the bases of support for the participant. None of the participants passed the highest level of the game.The second game was designed to stimulate rotations of the head and upper body. To this end, we designed a task wherein the user had to track and point at a moving ball with a virtual laser pointer for some amount of time until the ball would burst. Figure 1 (right) illustrates the spatial relationships and interaction between user and the virtual objects. As above, at the start of each game, a WCS is positioned 2 m in front of the user’s head position. The movement of the ball consisted of piecewise linear trajectories in the xy-plane of the WCS with constant velocity (*v)*. This trajectory would start with a random position and direction and bounce off with random (not physically correct) angles whenever the border of the motion area was approached. The extent of this motion area was determined by the horizontal (α_h_) and vertical (α_v_) angles that this area subtends within the field of view of the observer. Attached to the LCS of the HoloLens is a virtual laser that points towards the negative z-axis. By that, control of the pointer direction is independent from the animation of the ball motion in the WCS. In the final version of the game, the laser was not visualized as a solid line (like illustrated in Fig. 1); instead, for visual clarity only a small white dot was used to indicate its direction within the field of view of the observer (see Fig. 2, right). We placed a mesh-grid a small distance behind the xy-plane in order to provide additional stereoscopic cues to the observer for better depth perception of the virtual scene objects. During the game, users had to fixate the ball with the laser for some predefined time interval until the ball would burst. When the ball was focussed on, a timer was started and the percentage of the time already elapsed was visualized using an increasing white arc surrounding the ball (see Fig. 2, right). When the ball was lost for only a short duration, the timer would be re-set. This second game was built for being used both in a standing pose and for locomotion along a straight path. Therefore, we added a tracking function to automatically move the WCS along the z axis (and only the z-axis) whenever the LCS would move along the z-axis. By this, the entire virtual game scenario would follow with the user moving in the real environment. The degree of difficulty for this task could be adjusted with six parameters including the velocity and size of the ball, the size of the area of ball motion in terms of visual angles α_h_ and α_v_, the duration of the timer, and the sensitivity of losing the ball in terms of a time interval. Table 2 gives an account of the parameterizations of the different game levels in our study. At the easiest level (Level 1), the ball moved slowly and mostly in the center of the field of view. At a higher level of difficulty, there was an increased spread of movement of the ball from the center of field of view and the ball moved faster. Again, the base of support was also used to increase the difficulty of training. Participants performed this game also walking along a corridor. Each participant trained for 20 min with the help of the training application using the HoloLens and with support from the physiotherapist. Support from the physiotherapist could consist of, for example, technical questions about the HoloLens and that the training was performed safely. Feedback from the HoloLens consisted of the following: to see how long it was left until the ball burst, a sound when you managed to burst the ball, an arrow showing where the ball is when it is not in the screen, and how many points one received during the game. Depending on how well the training went, i.e. how many points one received, the level automatically increased the difficulty of balance training. The distribution between the two games was equal. The training was repeated twice a week for six weeks.

**Table 1:** The parameterizations for game 1 at different levels of difficulty

Level	*v* [cm/sec.]	α_v_ [deg.]	α_h_ [deg.]	lapse [sec.]	count
1	20	0	6	10	10
2	25	0.4	8	9	10
3	30	0.8	10	8	10
4	35	1.2	12	6	12
5	40	1.6	14	5	12
6	45	2	16	4	12
7	50	2.4	18	3	15
8	52	2.8	20	3	15
9	54	3.2	22	3	15
10	56	5.6	24	3	15

**Table 2:** The parameterizations for game 2 at different levels of difficulty

Level	*v* [cm/sec.]	size [cm]	α_v_ [deg.]	α_h_ [deg.]	time [sec.]	Lost [sec.]
1	10	15	40	40	5	1
2	15	15	40	60	7	1
3	20	12	40	80	10	1
4	25	12	40	90	10	0.5
5	30	55	20	90	10	0.5
6	35	12	40	90	10	0.5
7	40	15	40	90	10	1
8	45	15	40	100	10	1
9	50	20	40	110	10	1
10	55	20	40	120	10	1

### Data collection

A semi-structured interview guide was used where participants (Additional file 2) and physiotherapists (Additional file 1) were interviewed about the new hologram-based methodology for visual-interactive (AR) activity training for the older adults and about its feasibility.

Before and after the training period, balance was measured with a force platform Ergopower Technology (Porsgrunn, Norway). The force platform was connected to a personal computer via an analogue/digital converter (MuscleLab Model 4000e). The MuscleLab 8.0 software was used for calculations. The total sway (mm) during 30 s. of the center of pressure was calculated. The sampling interval was 0.01 s. (100 Hz). This procedure follows recommendations by Browne and O’Hare [26]. Participants measured their balance in four different positions (shoulder width, feet together, semi-standing and tandem standing with their feet) at 30 s. standing on a force platform. This was repeated twice, and an average of the two tests was calculated and used in the analysis. The instruction to the participant was to stand as still as possible and to fix the eyes at a point on the wall about two meters in front of the participant that was just below eye level. After this, participants conducted the Berg’s balance scale (BBS) [27, 28] and the Short Physical Performance Battery -Swedish version (SPPB-S) [29]. Finally, participants estimated their fear of fall with Falls Efficacy Scale Swedish version (FES-S) [30] – with a higher score on the scale indicating a reduced fear of falling. One participant could not complete the intervention because of illness**.**

### Data analysis

The first author (SB) performed all interviews with the participants. Afterwards, the interviews were transcribed verbatim and read through several times to ensure understanding of and familiarity with the text. The text was divided into meaning units based on the study aim, and was thereafter condensed and coded. The codes were then compared for similarities and differences and compiled into categories based on similarities. For an example of the data analysis process see Table 3. Analysis was done by the authors SB and ME. To increase ‘trustworthiness’, recommendations by Graneheim et al. [31] were followed. We tried to increase credibility by putting together a group of different participants, by using open questions, and by describing the method carefully. In order to meet the transferability criterion, we tried to describe the data and have the participants cited in the text. We also tried to fulfil dependability and credibility criteria in that the first author (SB) did the whole procedure of dividing into meaning units, condensing, codeding, and then sorting data inductively into categories. This procedure was monitored and discussed by one of the other authors (ME) to reach agreement in how data were handled.

**Table 3:** Examples of meaning-bearing units, condensed, codes and categories. I = interviewer, X = participant

meaning units	condensed	coded	categories
I: How did you think the technology worked in general? Was it a lot of trouble, or did it work well? X: No, I do not think it was too much trouble. Absolutely not. It was simple. As long as you got the glasses to sit, it was good. I think so.	Absolutely not any trouble. It was easy as long as you got the glasses to sit.	It was easy as long as you got the glasses to sit.	Usability of new technology

### Ethical approval and consent to participate

All participants were informed orally and in writing about the study purpose, about what it meant to participate, and about confidentiality. All participants stated that they understood the information and signed a written consent to participate. Participation was voluntary and the data were treated confidentially. The study was approved by the Regional Ethical Review Boards in Uppsala, Sweden (Dnr 2018/357).

## Results

The reasons for balance impairment varied among the participants from pain and surgery to various diseases and medication. During the baseline assessment, all participants stated that their balance impairments had caused them to be less active and to cut down on activities like chores, walking up stairs, climbing ladders, cycling, cleaning, going out in the winter, etc. Many experienced dizziness when changing positions, e.g. getting up from bed or a chair, turning around, etc. Three participants had fallen, and one of these had fallen several times. Six out of seven participants experienced a fear of falling due to their balance impairments. The reasons for taking part in the study varied, but all had hoped that their balance would improve. Participants also expressed that it would be fun, interesting, and exciting to take part. During the study, no falls occurred to any of the participants.

### Practicality around balance training

Six participants thought that exercising 15–20 min was adequate and was not too long. One participant expressed that it could have been even longer. Five participants experienced that training twice a week at fixed times was good, and it was easy to plan. However, two participants felt that they were ‘stuck’ with the fixed training times, which made it difficult for them to do anything else like travelling to their summer cottage. These participants expressed that they preferred to exercise more often but not so long since this would have a better effect on the training, “*… .probably best to do it a little more often. This would give better results; perhaps every other day or so;” participant 2.* This opinion was also shared by the physiotherapists who thought that the training could be a bit too long for some. Some participants would get tired, experience pain and lose concentration. The physiotherapists felt it best that participants would train a bit less but more often, *“… .when you are going for such long intervals, one gets tired in the body also. Then it is not just the balance that becomes an obstacle, but tiredness also occurs in the hip muscles and in the feet [ …*.]. *It is better not to push further but instead use shorter intervals more often;” physiotherapist 2.*

### Experiences of the two training games

#### Training game 1

Four of the participants had difficulty moving the center of gravity to the side. All felt that the exercises went well in the beginning when the ball did not move so much to the side and the base of support was large. When the ball went more to the side and moved faster, four participants had problems moving the center of gravity to the side, and especially when the base of support became smaller. Five participants felt that the ball disappeared from the screen. They also experienced that the exercise became static and predictable as the balls would alternately come from the right and left, “*… .you could catch it if you moved your feet; if you twisted your head the ball would disappear;” participant 5,* or” *…*. *it felt like I was playing tennis and that was fun, but in the end it became a bit tedious […*]; *at the same time you feel that it is useful for the hips to do that movement;” participant 4.*

The physiotherapists had similar experiences of the exercise and found it was difficult to move the weight between feet without turning the head at the same time. As a result, the participants turned their heads, and the ball disappeared from the screen. The physiotherapists considered this exercise to be a difficult motor performance. As a result, many did not manage the exercise when demands increased, which did not lead to a good balance exercise. The physiotherapists also experienced that the exercise was repetitious and boring, which led to reduced motivation among the participants,” *…. it was a complicated move to do because the participant would lean in one direction making it difficult with a rotation of the neck in the other direction to keep the glasses in the same plane. It was a motor challenge that was very difficult for the participants;” physiotherapist 2.*

#### Training game 2

Participants expressed that they had to concentrate a lot to be able to follow the ball all the way until it burst. There was no problem to follow the ball when standing still, however, it became more difficult when the base of support decreased or when participants walked in the corridor. The ball could disappear from the screen but participants were given information about where the ball was via feedback by an arrow. Five participants felt that walking was more positive than standing still and following the ball, “…. *I thought walking in the corridor went well, it was fun; the only problem was that turn. I stopped and didn’t know where the ball went” participant 4,* or *“…. it was kind of fun walking like this; it is monotonous when you stand still in the room;” participant 6*. The physiotherapists thought training to follow the ball worked well, and that the training could expand and increase in difficulty in the future. They also felt, as participants did, that it was more positive to exercise walking in the corridor than standing still.

The physiotherapist wanted more exercise programs to choose from in order to increase the motivation and concentration of the participants. They also pointed out that the participants needed to understand that they had to challenge themselves when training to get good effects. Also, clear goals for the participants with the training were needed. It was not just the figures indicating they were getting better balance during a test, but also a desire to see this in everyday life. This was a way to increase participants’ motivation, *“…. some say, ‘no, but I do not dare stand like that because I get so wobbly’. But then you have to remind them again that it is a balance study, and that it is the balance we are challenging. They cannot stay in their safety zone because then they do not improve. [ …]. They have to push to the point of being a bit uncomfortable, and stretch the limits in order to get better. ;” physiotherapist 2.*

### The increasing difficulty level of balance training

None of the participants experienced that the increase in the severity of the exercise was too fast, e.g. reducing the base of support, but none managed to train on one leg, *“…*. I *really thought it was fun. I didn’t really feel it increasing;” participant 5,* or *“…. I cannot manage with only one foot; I had to have the toes of one foot on the other [ ….]; otherwise it went well;” participant 1.*

### Feedback from the HoloLens to the participants during training

Five participants felt that feedback on how the training went, based on how many points they received, was positive, but they wanted to know more clearly what the points stood for and which points were good or bad, *“…. I did not really understand if it was counted all together or if it was one moment at a time;” participant 4.* Five participants also thought that the feedback from the technology could be motivated to boost one to train a little more, *“…. It is difficult to get people to work out, but when you are doing something like catching balls, it can be justified. It’s like a fun game;” participant 5,* or” …. *I thought several times that, now I have to start a little more with the waist like this and stretch out, and then I can take that ball [...]; this is something you get from the technology, and in this way it has been very good;” participant 4.*

Other feedback that participants experienced as positive was that one could see where the ball was by looking at the arrow on the screen, and also that you could see how far you had left before the ball burst by looking at the circle around the ball, *“… .then the ring around the ball would start growing. Then when it merged, there was a bursting [ …*] *then one had succeeded, and it was just to find the arrow again and start with the next ball;” participant 5.* There were mixed opinions about what the participants felt about audio feedback. Some felt that the sound did not really match what they experienced, while others thought it was good that they received feedback through sound when they managed to catch the ball.

### The experience of the physical and technical aspects of the HoloLens and its application

Six participants felt that wearing the HoloLens was not a problem, although some thought it was heavy to wear, *“…. It felt a bit heavy because I have neck problems. You can feel a little wobbly and not so steady [ …]; otherwise there was* no problem*;” participant 6*. All participants felt that the fit on the HoloLens was poor and it affected the training. Two participants got pain from the HoloLens either on the nasal root or on the forehead because the device needed to be tighten securely on the head in order for it to sit still, *“… I had to tighten it quite hard for it to sit still, so I got a bit sore on the nose;” participant 5.* The physiotherapists also perceived that it felt heavy and it pressed on the root of the nose and the forehead due to the tightening to make it sit still, *“… many complained that it was a pain having it on their nose, and they usually had quite a hard time setting it right. You can wiggle it up and down a bit and adjust tension;” physiotherapist 1.* The physiotherapists also came up with suggestions on what could improve the fit, e.g. some type of padding for the nose or some type of helmet insert so that it rests on the head and not only on the root of the nose and forehead.

Three participants felt that it would be good if even the person who helps with the training could also see what is happening on the screen in the HoloLens. This would make it easier to explain and to provide feedback to the user, which would facilitate the training, *“… .then you had someone to communicate with [ ….]; this, for example, is how it should be;” participant 1.* To facilitate in the future, the physiotherapists thought it would also be helpful if the person next in line could see and hear the same thing as the person who trained with the HoloLens. This would make it easier to correct the training and to provide feedback, *“…* .*had one been able to see and control things from the side, that would be all the feedback you needed. Then it would have been optimal;” physiotherapist 2.*

The HoloLens was calibrated at start-up. Many experienced this as a sensitive moment. If the HoloLens did not sit well on the head and you changed it, the calibration of the screen could also be changed. Then there was a need to recalibrate in order to continue training. This took time and was annoying. Sometimes when a participant turned their head in a certain position, what was seen on the screen could be affected. In some cases, what was on the screen became half or disappeared completely. This made it impossible to follow or to catch the ball, which many felt was frustrating, *“… .sometimes I thought I would tighten it a little more, but then it would slide downwards [ ….]; then I would not want to change it because usually the screen itself also changed;” participant 1.* The physiotherapists experienced a lot of trouble with the technique. It was difficult to get the HoloLens in the right place on the participants’ heads so that they could see the entire screen. The HoloLens had difficulties calibrating the environment so it became correct, and it took a long time to calibrate. This could cause the game pitch to be slightly shifted, and the participants would have difficulty completing the training. Sometimes the game needed to be recalibrated during the game itself, which took time and created frustration. The hand and finger commands that controlled the game were difficult to execute and were sensitive to both the physiotherapists and participants. As a result, it took a long time, for example, to start the game or to change the exercise program. It was difficult to maneuver and reverse in the menus because these were not user-friendly. The physiotherapists felt the screen was small and that participants could not see that much in the field of view; this made it difficult to see things from the sides and upwards/downwards, *“… now it is just a small part of the field of view that is affected. If the screen was bigger in the future, it would open up possibilities such as you could see things coming from the side;” physiotherapist 1.* Contrast on the screen was also something that was pointed out, i.e. that the HoloLens was good at seeing bigger things but harder with smaller objects. Some suggestions came up on how to improve the technique. For example, having some kind of sight reference so that one would know that you had the HoloLens right. The calibration would be as in a ‘follow a ball’ game where the HoloLens calibrates itself continuously and not with a fixed calibration such as in the game ‘to catch a ball’. Another suggestion was to be easier to move back in the menus with a back button, *“ …. you would need some kind of sight-reference points or lines, or something like that when you put on the glasses [ …]; you could put points in the corners of the screens so you could see all four points. If you did not see it, then you may have to make changes;” physiotherapist 2.*

### Clarity

Clarity was the category that emerged regularly during the interviews. This referred to everything from clear instructions to what the training was about and how to start the program. Also, clarity on how the training should be performed, about what the score stands for, and about when you are successful in the training was desired, *“…*. A*t first I thought you couldn’t move your head, but then you were allowed to move your head. Then what to do with the hands [ ….] was something that could have been more clear;” participant 1.*

### Exercise at home

Six participants were positive that they would consider practicing balance exercises at home using the HoloLens, and most felt that they could recommend to friends to train with the help of the HoloLens. They did not feel that the technology would be a major obstacle to training, but two participants thought that motivation would be, and that the social part would disappear when exercising at home. In order to increase motivation, some participants felt that someone needed to follow-up the training, and here the technology could be helpful, for example, to be able to see how much you exercised and the progress you made with the training, *“ … .this would not be a problem for this technology [ …]. If you notice improvement, then you might be more motivated;” participant 1.* Both physiotherapists mentioned home exercising and they saw opportunities in the future. Technology can be helpful with clear instructions via video and sound as well as increasing and progressing training. It can also help motivate and enable compliance to training, which could lead to better training results,” *…. It is compliance to maintain it over time, that is the problem […]; many say they need a little ‘carrot and stick approach’ sometimes. If you go home with the understanding that, ‘If you do not exercise they will be able to see that’; many perceive this as a strong motivation;” physiotherapist 2.* The physiotherapists experienced the HoloLens as easy, convenient, relatively safe, and cost-effective to train with. It is fun and it becomes more like a game than training, which can be motivating, *“… .first of all, it is fun to play a game and get training as a benefit [ ….]; you know how to get immersed in games and you can be motivated [ ….]. It can be easy to have training regularly, daily in the home environment. It is both time- and cost-effective;” physiotherapist 1.*

### Usability of new technology

Overall, the technology worked well and six out of seven participants were positive that they would be able to train at home with the HoloLens. The physiotherapists felt that there would be no major problems for the older adults to train with the new technology. In addition, it is an advantage if the person has an interest in technology when learning the new technology, “… .*for a normal person there should be no problems to learn the technology;” physiotherapist 1.* However, there were some parts of the new technology that the older adults had difficulty with such as movement with their hand and fingers to control the program or being able to maneuver in the menus on the screen. The sight dot was also difficult to see.

### Effects on the balance of exercise

When asked if they felt that their balance had changed after the training period, four participants replied that they felt they had become steadier, *“…. I feel that I have become more stable with the training, although it was an exercise that I could not have imagined since it was not possible to catch the balls anyway (with laughter);” participant 5.* Two participants did not experience any change, and one participant experienced a decline – she described that she became dizzier due to changing medication.

### Balance ability and fear of falling

**Fig. 3:**
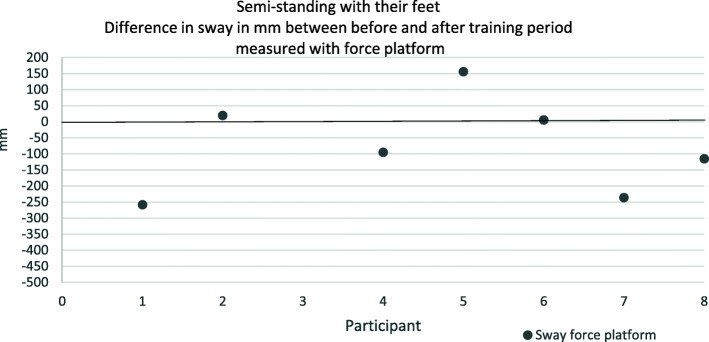
A dot below the zero-line indicates the participant has reduced his sway during the training period. A black dot represents sway measured with Force platform

**Fig. 4:**
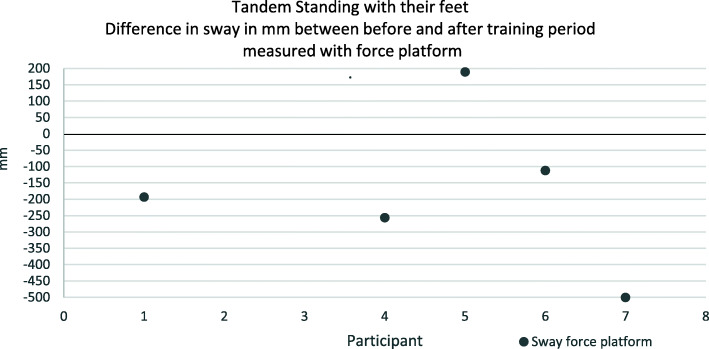
A dot below the zero-line indicates the participant has reduced his sway during the training period. A black dot represents sway measured with Force platform

Differences in sway before and after the training period can be seen in Figs. 3 and 4. For sway measured with the force platform semi-standing with their feet, two participants increased their sway numerically and four participants decreased their sway numerically after training. For tandem standing one participant increased the sway and four participants decreased the sway after training. It is noteworthy to point out that participant eight managed to stand tandem for 30 s. after the training period, which she did not do before the training. Participant two failed to do tandem standing and participant three had to withdraw due to illness. The self-assessment instruments showed no clear results (Table 4). An overall summary of the balance tests is that there was no improvements in balance after training.

**Table 4:** Participants’ points on the Berg Balance Scale (BBS), Performance Battery -Swedish version (SPPB-S), and the Falls Efficacy Scale International (FES-S) before and after the training period

Participant	BBS before	BBS after	SPPB-S before	SPPB-S after	FES-S before	FES-S after
1	53	51	11	11	123	130
2	50	49	8	9	122	128
3	–	–	–	–	–	–
4	53	55	12	12	130	130
5	56	55	9	11	86	104
6	56	49	11	11	100	127
7	52	51	10	10	62	77
8	49	50	12	9	120	108

The maximum scores for BBS are 56 points and a higher value indicate better balance ability, for SPPB-S 12 are maximum scores and higher value indicate better function of the lower extremity and for FES-S are 130 maximum score and higher value indicate lower concerns about falling

## Discussion

The purpose of this study was to test the feasibility of a new AR-based methodology for visual-interactive activity training for the older adults with balance impairments. The interview results showed that most of the participants experienced the new technology as positive. This was in regard to the training programs, increasing motivation, feedback technique, and design of the HoloLens and its application. However, there was room for improvements. For example, training game 1 was difficult to perform, and when the difficulty level increased, it was monotonous. The HoloLens was perceived as heavy, difficult to control the application with the various hand movements, and problematic to switch between the different menus in the application; also, the calibration took a long time. The physiotherapists felt that it was not a major problem for the older adults to handle the HoloLens as long as participants have no cognitive impairment. Four out of seven participants experienced that they become steadier after the training. Results of the balance tests showed no improvements in balance performance after AR training.

In a literature review, Tsertsidis et al. [32] found that when the older adults had difficulty in using technology, this was due to technical and design problems. Our participants also had these experiences and they were not satisfied with the way the application was designed, which meant that it was difficult to control the application. Furthermore, the older adults expressed that the technology in itself created problems that could lead to frustration, e.g. calibration took a long time. Therefore, it is important to change the design of the application in the HoloLens to be more user-friendly for the older adults. One example is by making it easier to navigate through the various menus perhaps with the help of handheld clicks so that the older adults do not have to use the various hand manoeuvres that control the application. Also, the calibration of the HoloLens must be easier and more stable.

The participants also experienced the benefits of the new technology such that it could increase motivation, it was fun to train with, and it could help to show participants how to train. This is consistent with Tsertsidis et al. [32] who found that the older adults saw benefits of the technology such as increased awareness of health condition, increased independence, and increased enjoyment.

Several of the participants experienced training game 1 difficult. They found it hard to move the center of gravity to the side, because when they did, the ball disappeared out of the field of view if they did not simultaneously turn their head so as to look straight forward towards the approaching ball (compare Fig. 1, left). Even physiotherapists experienced this game to be a difficult motor performance. As a result, only few managed the exercise at higher levels where demands increased according to the parameter choices in Table 1, which did not lead to a good balance exercise. This problem is inherent to the way in which the game logic and spatial interaction in game 1 is designed, where the “ring” is locket to the line of sight of the viewer and therefore head rotation is not instrumental to catch a ball in sideways motion. To alleviate the problems encountered by test participants, the degree of difficulty of the game should be adjusted, which foremost requires lowering the maximum horizontal angle α_h_ at which the ball approaches the observer in all levels of the game.

Although many of the participants did not experience the training as too long (15–20 min), some participants, as well as the physiotherapists, felt that the training time was long and it was easy to lose motivation and concentration. Their suggestion was to train more often but for a shorter period of time to get a better focus on the training. In a systematic review and meta-analysis by Lesinski et al. [33], it was recommended that the training should be between 30 and 45 min per session and the total training duration per week between 90 and 120 min to have an effect on the balance ability. The reason that the participants in our study lost motivation and concentration may be because the training consisted of only two exercises, which some experienced as static, predictable and monotonous, and thereby caused them to lose concentration. Therefore, the next step in the development of the new technology will be to introduce new exercise games to avoid monotony and to increase motivation, but also to exercise different parts of the balance ability.

Feedback and motivation were raised by many participants during the interview as something that was positive with the new technology. This applied both to doing the exercise correctly and to giving a little more effort to get an extra point or go to the next level in the game. The physiotherapists also felt that feedback from the technique stimulated participants to exercise and to try a little harder. Research has shown that in order for the training to be effective, one must follow the training advice [9, 10], and a study showed that many older adults find it difficult to fully follow the training advice they received during home training [11]. In this regard, the new technology can be helpful for the older adults who are exercising balance, not only to carry out the training but also to get help with progression and increase their motivation. This could increase the efficiency of training but as some participants expressed, feedback must be understandable, i.e. knowing what a good result is, what a poor result is, and what this means for one’s balance ability. This feedback is important to develop in the new technique to increase the understanding of those who exercise and thereby increase motivation.

Our study did not show any improvements in the balance tests after training for six weeks with the HoloLens, but the focus of this pilot study was to investigate feasibility of the intervention. However, the MRC recommends to include the intended tests/instruments to be used in the final study to see if they are suitable for the target group. In order to be able to evaluate effects of the intervention, the design needs to be developed to include more participants and a control group. The steps in this process is in accordance with recommendations for the development of complex interventions from the MRC [24, 25]. The next step in developing this training method is to improve the technique and extend the exercise games, which will then be tested in a new feasibility study. This new feasibility study should be combined with increasing training length (three months), frequency (2–3 times/week) and include multiple types of exercise all according to the recommendations of balance training for the older adults [1, 4, 7, 8]. According to MRC several feasibility studies might be needed before proceeding to the next step and testing the effects.

It is important to take into account that the study has limitations. The goal of the feasibility study was to discover the advantages and disadvantages of the study design in order to develop a better design before making major interventions according to the MRC [24, 25]. Our design was that participants trained at the health center in close cooperation and with the physiotherapists who also had been involved in the development of the new methodology for visual-interactive activity training. This may have affected participants’ experiences of the new technology. There could be several reasons why the study did not show any effect on the balance tests. For example, the time the group trained was too short to have effects on the balance and the number of participants was small; therefore, it is difficult to generalize and have sufficient statistical power to evaluate the intervention’s effectiveness. Therefore, the results should considered descriptive rather than hypothesis testing, the exercises in the pilot study did not contain all the parts that the balance training should contain [1, 4, 7, 8], and it could simply be that the training with the HoloLens had no effect on the balance of the older adults. Although the number of participants was small, it is a strength that we used a mixed-method design and validated measuring instruments, and we interviewed both the participants and the physiotherapists. It is important to note that there may have been bias in the results when recruitment was voluntary and that the two physiotherapists who recruited and trained the participants did the training and assist with some of the tests. Therefore, the results of this pilot study should be treated with caution.

## Conclusion

The study showed that the new hologram-based methodology for visual-interactive activity training for the older adults is to some extent feasible. The technology seems to be able to stimulate increased motivation, which is a prerequisite for adherence to training. No improvements in balance performance after AR training could be discerned, but the used balance tests seem to be feasible for this target group. However, more development is needed; and after that, a new feasibility study is required before undertaking a larger and longer intervention study.

## Supplementary Information

**Additional file 1.**

**Additional file 2.**

**Additional file 3.**

## Data Availability

The datasets during and/or analysed during the current study are available from the corresponding author on reasonable request.

## Abbreviations

AR: Augmented Reality
BBS: Berg’s balance scale
FES-S: Falls Efficacy Scale - Swedish version
FoV_h_: Horizontal field of view
Hz: Hertz
LCS: Local Coordinate System
MRC: Medical Research Council
*v*: Velocity
SPPB-S: Short Physical Performance Battery -Swedish version
WCS: World Coordinate System
VR: Virtual Reality
α_h_: Horizontal angular ranges
α_v_: Vertical angular ranges
