# Supplementary material for: Using augmented reality technology for balance training in the older adults: a feasibility pilot study
Source: BMC Geriatr. 2021 Feb 26;21:144. doi: 10.1186/s12877-021-02061-9 (PMC7913413; doi:10.1186/s12877-021-02061-9)
Supplement: Supplementary file 1 — Additional file 1. [file 12877_2021_2061_MOESM1_ESM.docx]

Enclosed are a video clip from the HoloLen game 1 and 2 and a video about the training. The latter video is in Swedish, but you can still get an understanding of how the training works.

Game 2 <https://youtu.be/_C-ZqU1wxe0>

Video in Swedish <https://youtu.be/ZQSQUQqMq38>

Game 1 <https://youtu.be/tFypxjJk-dE>
