# Supplementary material for: Using augmented reality technology for balance training in the older adults: a feasibility pilot study
Source: BMC Geriatr. 2021 Feb 26;21:144. doi: 10.1186/s12877-021-02061-9 (PMC7913413; doi:10.1186/s12877-021-02061-9)
Supplement: Supplementary file 2 — Additional file 2. [file 12877_2021_2061_MOESM2_ESM.docx]

**The physiotherapists’ interview guide. This will be more refined during work on the research project. In addition, pilot interviews will be done before the final interview guide is produced. The overall subject areas are as follows:**

Tell us about your previous experiences with balance training for the elderly.

When you heard about the project and the AR glasses, what were your first thoughts? In addition, how was it when you started testing?

What are your thoughts about how the elderly complied with the new technology?

- What did you feel they had difficulty with and worked well with regarding the new technology?

Tell us about your experiences with the new technology.

- What do you think are the disadvantages of the new technology and balance training for the elderly?
- What do you think are the advantages of the new technology and balance training for the elderly?
- What improvements are needed to the technology?
- What else can be done better?

Tell us about your experiences of the balance training.

- What improvements need to be made to balance training?
- What about the frequency, duration and length of training?

Tell us what you think about the new technology as an aid to balance training for the elderly at home.
