# Supplementary material for: Using augmented reality technology for balance training in the older adults: a feasibility pilot study
Source: BMC Geriatr. 2021 Feb 26;21:144. doi: 10.1186/s12877-021-02061-9 (PMC7913413; doi:10.1186/s12877-021-02061-9)
Supplement: Supplementary file 3 — Additional file 3. [file 12877_2021_2061_MOESM3_ESM.docx]

How would you describe your balance?

- What situations in your everyday life are affected by your impaired balance?
- When is your impaired balance an obstacle for you?
- In what way does your impaired balance affect you?
- Are you afraid of falling?
- Have you fallen because of your impaired balance?

When you heard about the project and the AR glasses, what were your first thoughts?

- What feelings did you experience when you got the request to be part of this project?
- Why did you feel that way you did?
- Why did you agree to be a part of the project?
- Did you get enough information about the project, or was something missing?

Tell us about your experiences with the new technology.

- Did you receive sufficient information about the technology to be able to use the equipment
- Ask about the process - every step if they do not tell
- Tell us about it, and how it was when you first started testing.
- What was it like to wear the AR glasses?
- How did the AR glasses fit?
- How do you think the technology worked in the AR glasses?
- What was it like to follow the oral instructions that were given through the AR glasses?
- What was it like to follow the visual instructions that were given through the AR glasses?
- What are your thoughts about the feedback you received from the AR glasses when you trained?

Tell us about your experiences of balance training.

- What do you think about training with the help of the AR glasses?
- What are the disadvantages of training with the AR glasses?
- What are the advantages of training with the AR glasses?
- What was it like to do the training programs when you had to follow the object with your eyes using the AR glasses?
- What was it like to perform the training programs when you would capture and avoid objects while standing and using the AR glasses?
- What was it like to perform the training programs when you were walking in the corridor and performing various exercises using the AR glasses?
- What do you think about the increasing levels of difficulty in your training program?
- What do you think about the scope of the balance training?

What are thoughts about the practical aspects of the balance training?

- What do you think about going and training at the health center twice a week?
- What about the transportation?
- What about the extent of the frequency and duration of training?

Tell us what you think about training with the new technology in your home environment.

- Would you be able to train with the AR glasses without the help of a physiotherapist?
- Would you be able to train at home with the AR glasses without help?
- Were there any instructions missing such to interfere with you train yourself with the AR glasses?
- Is this type of exercise something that you would recommend to your friends who have impaired balance?

Has your balance changed during the training period?

- Describe in what way.

Is there something else you would like to tell us about that has not already been addressed?
